# Specific TATAA and bZIP requirements suggest that HTLV-I Tax has transcriptional activity subsequent to the assembly of an initiation complex

**DOI:** 10.1186/1742-4690-1-18

**Published:** 2004-07-30

**Authors:** Yick-Pang Ching, Abel CS Chun, King-Tung Chin, Zhi-Qing Zhang, Kuan-Teh Jeang, Dong-Yan Jin

**Affiliations:** 1 Department of Biochemistry, The University of Hong Kong, Pokfulam, Hong Kong, China; 2 Department of Pathology, The University of Hong Kong, Pokfulam, Hong Kong, China; 3 National Key Laboratory for Molecular Virology, Institute of Virology, 100 Yingxin Street, Beijing 100052, China; 4 Laboratory of Molecular Microbiology, National Institutes of Health, 9000 Rockville Pike, Bethesda, MD 20892-0460, USA

## Abstract

**Background:**

Human T-cell leukemia virus type I (HTLV-I) Tax protein is a transcriptional regulator of viral and cellular genes. In this study we have examined in detail the determinants for Tax-mediated transcriptional activation.

**Results:**

Whereas previously the LTR enhancer elements were thought to be the sole Tax-targets, herein, we find that the core HTLV-I TATAA motif also provides specific responsiveness not seen with either the SV40 or the E1b TATAA boxes. When enhancer elements which can mediate Tax-responsiveness were compared, the authentic HTLV-I 21-bp repeats were found to be the most effective. Related bZIP factors such as CREB, ATF4, c-Jun and LZIP are often thought to recognize the 21-bp repeats equivalently. However, amongst bZIP factors, we found that CREB, by far, is preferred by Tax for activation. When LTR transcription was reconstituted by substituting either κB or serum response elements in place of the 21-bp repeats, Tax activated these surrogate motifs using surfaces which are different from that utilized for CREB interaction. Finally, we employed artificial recruitment of TATA-binding protein to the HTLV-I promoter in "bypass" experiments to show for the first time that Tax has transcriptional activity subsequent to the assembly of an initiation complex at the promoter.

**Conclusions:**

Optimal activation of the HTLV-I LTR by Tax specifically requires the core HTLV-I TATAA promoter, CREB and the 21-bp repeats. In addition, we also provide the first evidence for transcriptional activity of Tax after the recruitment of TATA-binding protein to the promoter.

## Background

In eukaryotes, transcription by RNA polymerase II requires the orderly recruitment of basal transcription factors and activators to the core promoter and enhancers, respectively [1,2]. The core promoter contains the transcription initiation site, and it provides the docking sites for the basal transcription factors that nucleate the assembly of a functional preinitiation complex (PIC). The TATA box is one of four major core promoter elements, and it is specifically recognized by the TATA-binding protein (TBP), a subunit of the basal transcription factor TFIID which also contains at least 14 TBP-associated factors (TAFs). On the other hand, enhancers are bound by sequence-specific transcriptional activators that are thought to promote PIC assembly through interactions with components of the basal transcription machinery.

Human T-cell leukemia virus type I (HTLV-I) Tax protein is a unique transcriptional regulator [3]. Tax can modulate the HTLV-I long terminal repeats (LTR), heterologous viral promoters, and a variety of cellular genes. In most context, Tax acts as a potent transcriptional activator through Tax-responsive DNA elements that are recognized by cellular transcription factors CREB, NFκB and serum response factor (SRF) [4-6]. For activation of the HTLV-I LTR, Tax targets three imperfectly conserved 21-bp direct repeats flanked by GC-rich sequences. In this scenario, Tax forms a ternary complex with CREB and the 21-bp repeat through physical interaction with CREB and direct contact with the flanking GC-rich sequences [7-9]. Tax-induced activation of other promoters is thought to be mediated through protein-protein interactions. Thus, Tax is a pleiotropic transcriptional activator that targets multiple enhancer elements through multiple cellular transcription factors.

To date, the molecular mechanisms for Tax trans-activation have been well studied. Due to its pleiotropic activities, there are likely nuances to Tax's activity which remain unrevealed. Currently, we understand Tax to harbor a minimal activation domain [10], to interact with basal transcription factors such as TBP [11], to form a homo-dimer [12-14], and to stimulate the dimerization of cellular regulatory factors such as CREB [15,16] and IKK-γ [17]. Moreover, we also know that Tax can directly engage transcriptional coactivators such as CREB-binding protein, p300 and P/CAF [18-20]. However, it remains unclear what is Tax's optimal preference for an enhancer – TATAA configuration. It has also been unaddressed whether Tax has a transcriptional activity after the formation of an initiation complex at the TATAA-box.

In mammalian cells, the artificial recruitment of TBP sufficiently activates transcription from some promoters [21-24]. It is understood that the structure of core promoter is one important determinant for this activation [23]. On the other hand, DNA-tethered TBP can also work synergistically with selective natural activators such as human immunodeficiency virus type 1 (HIV-1) Tat protein [21-23] and cytomegalovirus IE2 protein [25]. In this regard, it is not known whether TBP recruitment suffices for activation of HTLV-I minimal promoter. Nor is it clear whether Tax can cooperate with promoter-tethered TBP.

Here, we have constructed a series of chimeric enhancer-TATAA reporters to analyze the functional roles of these transcription elements in Tax-mediated activation. We observed that Tax activates the HTLV-I 21-bp repeats more potently than other enhancer elements. Analysis of ten mutants of Tax revealed that Tax utilizes different domains to target different cellular factors. We also found that multiple bZIP transcription factors including the newly-identified LZIP are involved in Tax activation of HTLV-I LTR. Finally, two other salient findings are that optimal Tax-responsiveness is specified by the HTLV-I-specific TATAA element, and that Tax synergizes with artificially recruited, DNA-tethered, TBP in a phase of transcription after the assembly of an initiation complex at the promoter.

## Results

### Specific preference by Tax for only one enhancer element

Tax can activate transcription through 21-bp repeats, CRE, κB site or SRE [4-9]. However, a direct head-to-head comparison between the relative preferences of Tax for each of these elements is complicated by the context of additional DNA elements in the various promoters tested to date (i.e. the HTLV-I LTR versus the HIV-1 LTR versus the interleukin-2 promoter). To directly compare enhancer motifs, they should be placed in identical TATAA-context and tested in identical experimental settings. Towards this end, we constructed a series of six reporters to dissect the ordered preference of Tax for various enhancers.

**Figure 1:**
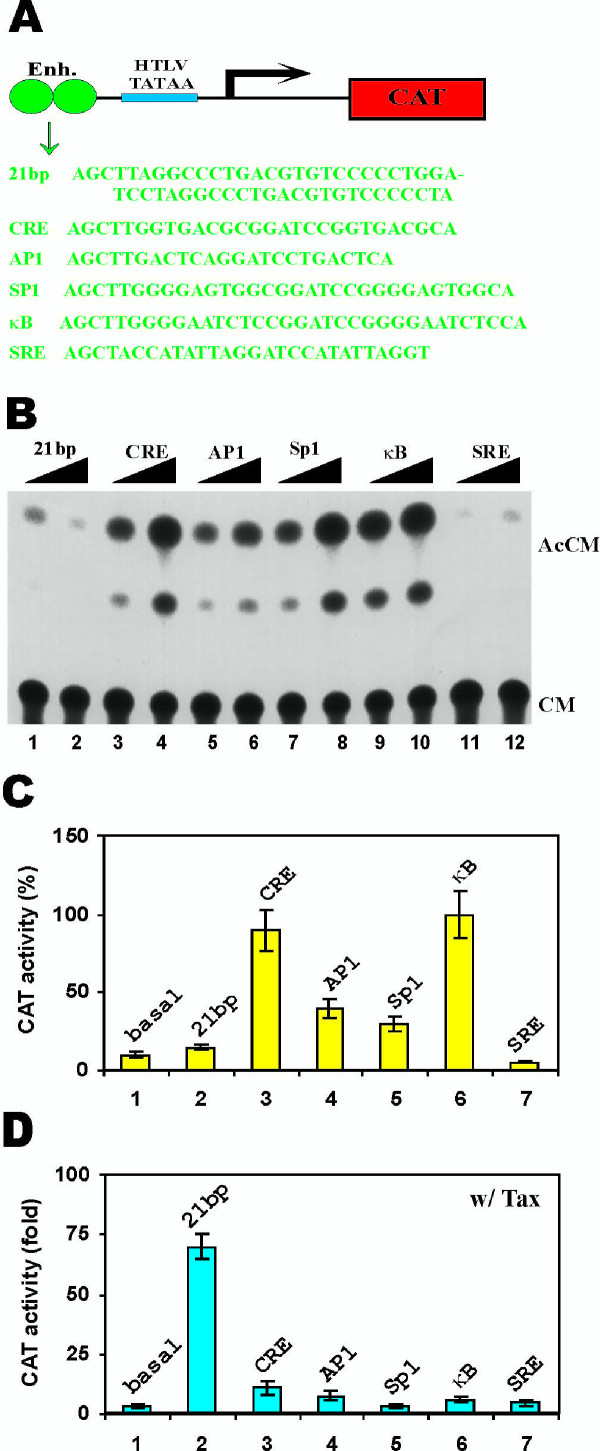
Relative responsiveness of enhancers to Tax in HeLa cells. (**A**) CAT reporter plasmid. Each plasmid contains two copies of enhancer elements (21-bp repeats, CRE, AP1, Sp1, κB and SRE) and one copy of HTLV-I minimal promoter (HTLV TATAA). The enhancer (Enh.) sequences are shown in green. (**B**) A representative example of CAT assay. Increasing amounts (5 to 10 μg) of p21-HTLV-CAT (lanes 1 and 2), pCRE-HTLV-CAT (lanes 3 and 4), pAP1-HTLV-CAT (lanes 5 and 6), pSP1-HTLV-CAT (lanes 7 and 8), pKB-HTLV-CAT (lanes 9 and 10) and pSRE-HTLV-CAT (lanes 11 and 12) were transfected into HeLa cells. CAT assays were performed 48 h after transfection. AcCM: acetyl chloramphenicol. CM: chloramphenicol. (**C**) Basal transcriptional activities of enhancer elements. Five microgram of plasmids containing the HTLV TATAA alone (pHTLV-CAT; column 1) or the indicated enhancer elements (columns 2 to 7) were transfected into HeLa cells and the relative CAT activities were compared. CAT activity from pKB-HTLV-CAT-transfected HeLa cells was taken as 100% (lane 6). (**D**) Tax-dependent transcriptional activities of enhancer elements. The same plasmids as in C plus 1 μg of Tax-expressing plasmid pIEX were co-transfected into HeLa cells and the CAT assays were performed. Fold activation in the presence of Tax versus in the absence of Tax was calculated and compared. All CAT results are representative of three independent experiments.

Each reporter contains two copies of enhancer motifs (21-bp repeats, CRE, AP1, Sp1, κB or SRE) and a minimal HTLV-I TATAA promoter (Fig. 1A). Because all reporters have the same HTLV-1 minimal promoter and are otherwise devoid of any known enhancer elements, side-by-side comparisons would reflect directly the contribution of the variously added cis-enhancer. We observed that the κB- and CRE- motifs had the highest basal activities in HeLa cells in the absence of Tax (Fig. 1B, lanes 3, 4, 9 and 10; and Fig. 1C, columns 3 and 6 compared to column 1). Of significant interest, in stark contrast to the cellular CRE elements, the reiterated HTLV-I 21-bp repeats (normally considered as viral CRE elements) and the SRE exerted little or no basal activity (Fig. 1B, lanes 1, 2, 11 and 12; and Fig. 1C, lanes 2 and 7 compared to lane 1). The AP1 and Sp1 sites were moderately active (Fig. 1B, lanes 5–8 and Fig. 1C, lanes 4 and 5). Hence for basal expression in the context of the HTLV-I TATAA promoter, κB, CRE > AP1, Sp1 >> 21 bp, SRE.

When the reporters were tested in the presence of Tax, a different pattern emerged. Transcription from the 21-bp repeats was stimulated approximately 70-fold (Fig. 1D, lane 2 compared to lane 1) while that from the Sp1 site, not prototypically known to be responsive to Tax, was not activated significantly over the activity of the HTLV-I minimal promoter (Fig. 1D, lane 5 compared to lane 1). All other responses to Tax were markedly weaker than that seen from the 21-bp repeats. Hence, for all practical purposes, only a duplicated 21-bp repeat in the context of isolated placement upstream of an authentic HTLV-I minimal TATAA box could be regarded as significantly Tax-responsive in HeLa cells.

**Figure 2:**
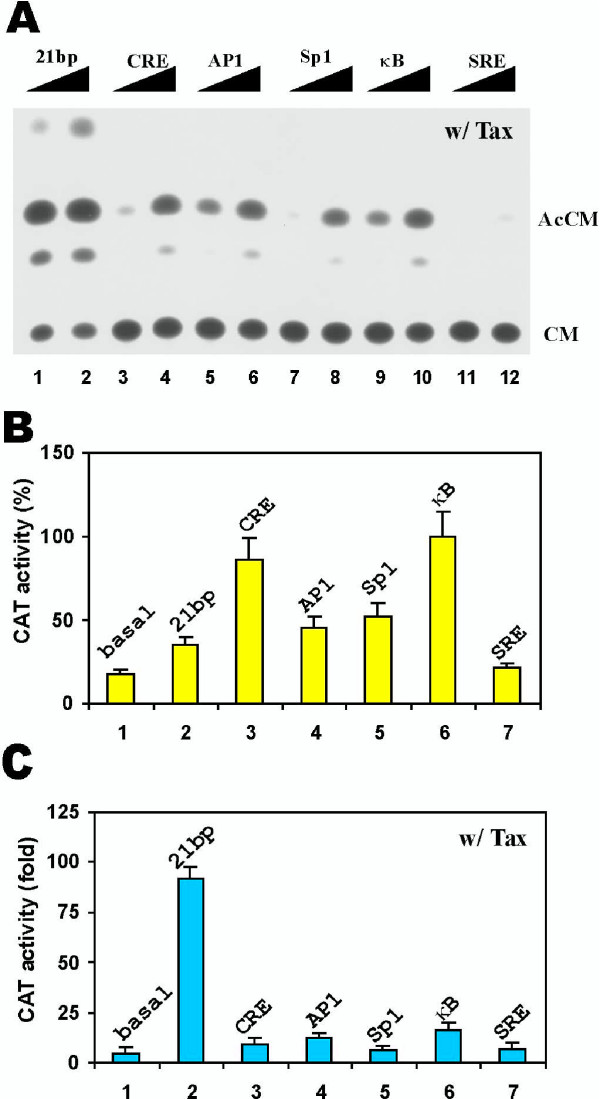
Relative responsiveness of enhancers to Tax in JPX9 cells. (**A**) A representative example of CAT assay. Tax-expressing plasmid pIEX (1 μg) and increasing amounts (0.5 to 1 μg) of p21-HTLV-CAT (lanes 1 and 2), pCRE-HTLV-CAT (lanes 3 and 4), pAP1-HTLV-CAT (lanes 5 and 6), pSP1-HTLV-CAT (lanes 7 and 8), pKB-HTLV-CAT (lanes 9 and 10) and pSRE-HTLV-CAT (lanes 11 and 12) were transfected into Jurkat cells. CAT assays were performed 48 h after transfection. AcCM: acetyl chloramphenicol. CM: chloramphenicol. (**B**) Basal transcriptional activities of enhancer elements. One microgram of plasmids containing the HTLV TATAA alone (pHTLV-CAT; column 1) or the indicated enhancer elements (columns 2 to 7) were transfected into Jurkat cells and the relative CAT activities were compared. CAT activity from pKB-HTLV-CAT-transfected Jurkat cells was taken as 100% (lane 6). (**D**) Tax-dependent transcriptional activities of enhancer elements. The same plasmids as in C plus 1 μg of Tax-expressing plasmid pIEX were co-transfected into Jurkat cells and the CAT assays were performed. Fold activation in the presence of Tax versus in the absence of Tax was calculated and compared. All CAT results are representative of three independent experiments.

We repeated the experiments in Jurkat T lymphocytes and obtained similar results (Fig. 2). Thus, while the κB and CRE enhancers displayed the highest activities in the absence of Tax (Fig. 2B, lanes 3 and 6 compared to lanes 5, 4, 2, 1, and 7), only the 21-bp repeats were highly responsive to Tax (Fig. 2A, lanes 1 and 2; Fig. 2C, lane 2). Our results from HeLa and Jurkat cells consistently support the preferential activation of the 21-bp repeats by Tax.

### Multiple activation surfaces are configured in Tax

In Fig. 1D, the 21-bp repeats were activated by Tax >75 fold, while κB and SRE motifs were activated five and three fold, respectively. The low activation of the latter motifs, although comparatively less significant than that from the 21 bp elements, was real and reproducible. To further understand how Tax works, we wondered whether the different magnitudes of activation were due to quantitative or qualitative differences in protein-protein interaction. To address this question, we examined the separate responses of the three motifs to a battery of Tax mutants.

**Figure 3:**
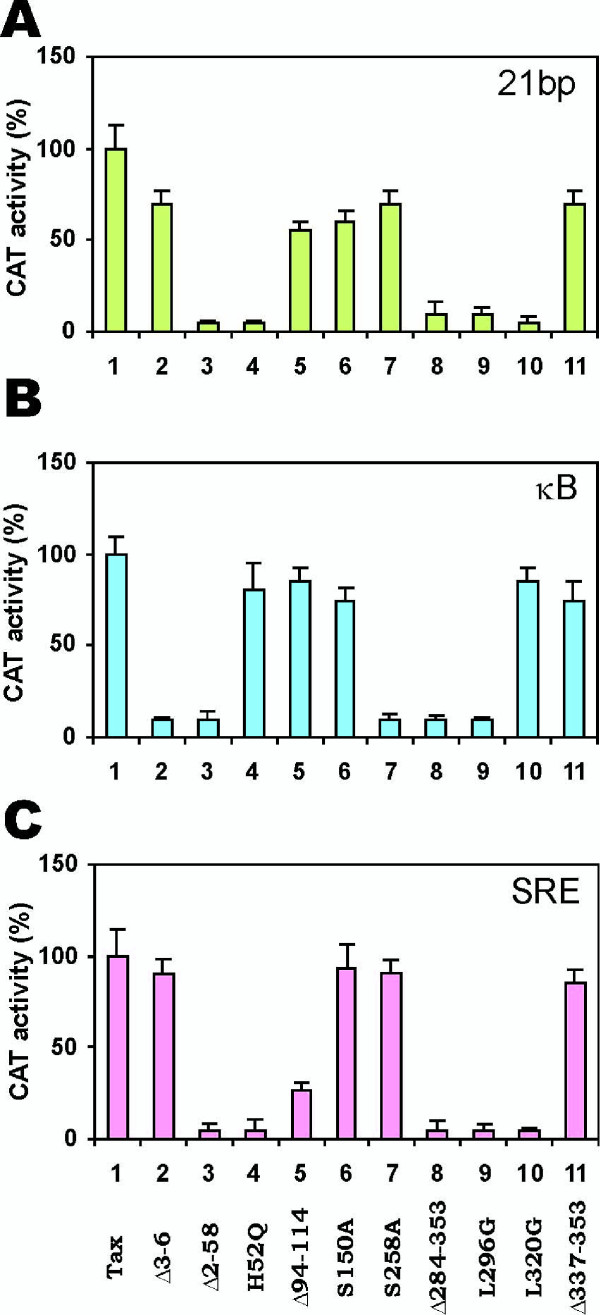
Differential activities of Tax mutants on 21-bp repeats (**A**), κB (**B**), and SRE (**C**) motifs. One microgram of plasmid expressing the indicated Tax mutants plus 5 μg of p21-HTLV-CAT, pKB-HTLV-CAT or pSRE-HTLV-CAT was individually transfected into HeLa cells. CAT activity from wild type Tax-transfected cells (lane 1) was taken as 100%.

Previously we had characterized 47 mutations in Tax that affect transcriptional activity [26]. Here we selected 10 of these Tax mutants to shed light on the discrete surfaces used by Tax to mediate effects on 21-bp repeats, κB and SRE. All mutants were expressed to comparable levels in HeLa cells (data not shown). Their relative activities on 21-bp repeats, κB and SRE were assessed (Fig. 3).

Based on percentage of activation relative to wild type Tax, we saw three patterns of mutant activity for 21 bp, κB and SRE (Fig. 3). Hence, the activation domain mutant Tax L320G [10] and the zinc finger mutant Tax H52Q [26] were defective in activating either 21-bp repeats or SRE, but were fully competent for κB (Fig. 3, lanes 4 and 10). By contrast, the N-terminal mutant Tax Δ3–6 and the point mutant Tax S258A activated 21-bp repeats and SRE well, but did not activate κB (Fig. 3, lanes 2 and 7). Additionally, mutants Tax Δ94–114, Tax S150A and Tax Δ337–353 were active on all three motifs (Fig. 3, lanes 5, 6 and 11), while neither Tax Δ2–58, Tax Δ 284–353 nor Tax L296G (Fig. 3, lanes 3, 8 and 9) activated any of the motifs. These non-identical patterns suggest that Tax may use different contact surfaces to target factors docked at the 21-bp repeats, κB or SRE. We note some similarity in the Tax mutant activity profiles for the 21-bp repeats and SRE suggesting that overlapping surfaces may be utilized.

### Amongst bZIP factors, CREB is specifically preferred by Tax

Tax activates the HTLV-I LTR through the viral 21-bp repeats [7-9]. When compared to κB and SRE, the activation of 21-bp repeats by Tax is particularly effective (Fig. 1 and Fig. 2) and, based on mutant profiles (Fig. 3A), relies upon unique structural surfaces. Previously, it has been proposed that bZIP cellular transcription factors including CREB [9,27,28], ATF4 [29,30] and c-Jun [31] play roles in Tax activation of 21-bp repeats. However, the relative contribution of these bZIP factors has not been compared directly in the same experimental setting. Furthermore, it remains undetermined whether additional newly identified bZIP proteins may also participate in Tax activation of 21-bp repeats.

**Figure 4:**
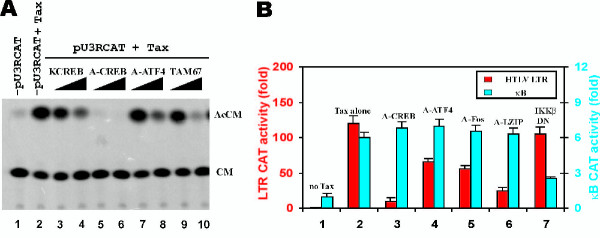
Specific preference for CREB by Tax. (**A**) An example of CAT assay. HeLa cells were transfected with pU3RCAT alone (lane 1), pU3RCAT plus Tax expression plasmid pIEX (lane 2) or pU3RCAT plus pIEX plus increasing amounts (5 to 10 μg) of plasmids expressing the indicated dominant-negative proteins (lanes 3–10). D-Threo-[dichloroacetyl-1-^14^C]-chloramphenicol was as used as substrate in the CAT assay. (**B**) Influence of dominant-negative proteins on Tax activation. The cells received pU3RCAT (red) or pKB-SV40-CAT (blue) only (group 1), pU3RCAT/pKB-SV40-CAT plus Tax-expressing plasmid pIEX (group 2) or pU3RCAT/pKB-SV40-CAT plus pIEX plus plasmids expressing the indicated dominant-negative proteins. The empty vector was used to normalize the amount of plasmids given to each group of cells. DN: dominant-negative.

We next used dominant-negative proteins to assess the contributory roles of different bZIP transcription factors on Tax-dependent activation. We employed several well-documented dominant-negative inhibitors of CREB and Jun proteins including KCREB [32], A-CREB [33], A-Fos [34] and TAM67 [35]. In addition, we constructed dominant-negative versions of ATF4 and LZIP [36] using the strategies suggested by Vinson et al. [37]. The dominant-inhibitory activities of the latter two proteins A-ATF4 and A-LZIP were verified using electrophoretic mobility shift assay and CAT reporter assay (data not shown). We interrogated these dominant negative bZIP proteins for inhibition of Tax activation of HTLV-I LTR (Fig. 4A). All, KCREB, A-CREB, A-ATF4 and TAM67, suppressed Tax activation in a dose-dependent manner (Fig. 4A, lanes 3–10 compared to lane 2). However, different dominant negative inhibitors constructed to the same protein using different strategies might have different potencies. For example, KCREB contains a mutation of a single amino acid in the CREB DNA-binding domain [32], whereas A-CREB was constructed by fusing a designed acidic amphipathic extension onto the N terminus of the CREB leucine zipper region [33]. Differential inhibitory effects of KCREB and A-CREB were observed (Fig. 4A, lanes 3–6). In light of this, we quantitated and compared the inhibitory activities of dominant negative proteins all constructed using the same strategy (Fig. 4B). Since NFκB is not involved in Tax activation of HTLV-I LTR, we included a dominant negative form of IKKβ, IKKβ DN, as a neutral control (Fig. 4B, group 7). When we compared four dominant negative bZIP proteins, A-CREB, A-LZIP, A-Fos and A-ATF4, constructed using the identical molecular strategy, we observed the most dramatic suppression of Tax activation of HTLV-I LTR with A-CREB (Fig. 4B, group 3, red column). The second most significant reduction in activity was seen with A-LZIP [36] (Fig. 4B, group 6, red column). Thus, although several bZIP proteins can redundantly serve to mediate Tax-activation of the LTR, a clear preference for CREB is revealed by our assay.

To verify the specificity of dominant negative effects, we also tested the activities of dominant negative proteins on an NFκB-dependent reporter (Fig. 4B, blue columns). Noticeably, none of the dominant negative bZIP proteins had an effect on Tax activation of NFκB (Fig. 4B, groups 3–6 compared to group 2, blue columns). In contrast, the expression of IKKβ DN led to more than 50% suppression of NFκB activity (Fig. 4B, group 7, blue column). These results ruled out the possibility that A-CREB, A-ATF4, A-Fos and A-LZIP might non-specifically inhibit transcription.

### Functional significance of the HTLV-I TATAA element to transcriptional activation by Tax

**Figure 5:**
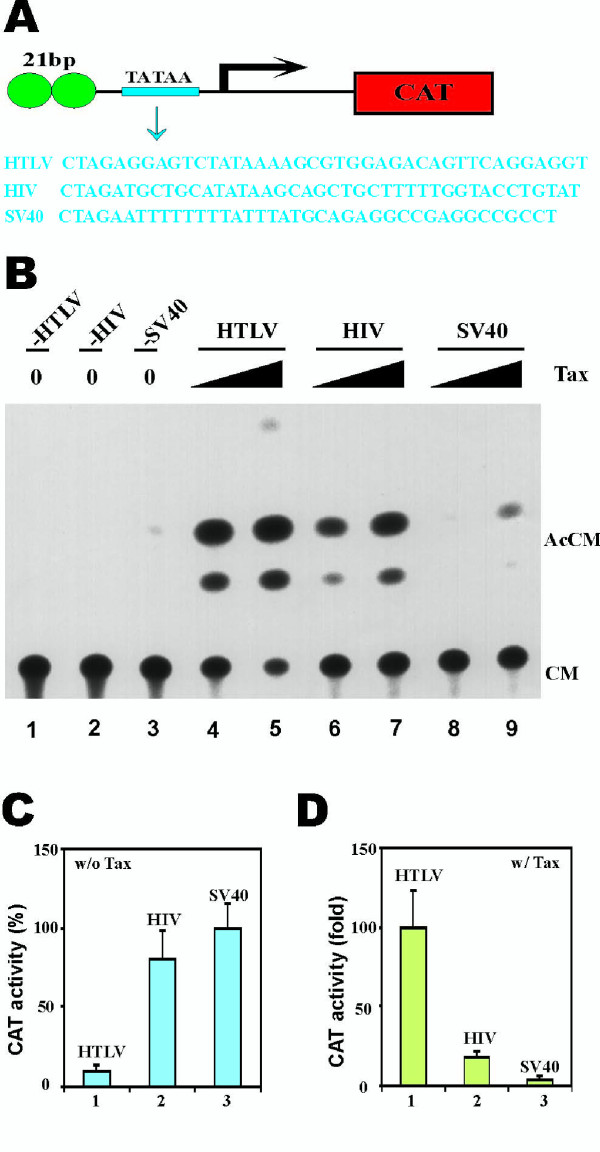
Tax preferentially activates the HTLV-I minimal TATAA promoter. (**A**) CAT reporter plasmid. Each plasmid contains two 21-bp repeats and one copy of minimal promoter (TATAA) from HTLV-I, HIV-1 and SV40. The minimal promoter sequences are shown in blue. (**B**) A representative example of CAT assay. The cells received 0, 0.5 and 1 μg of Tax-expressing plasmid pIEX and 5 μg of the indicated CAT reporter constructs (p21-HTLV-CAT, p21-HIV-CAT and p21-SV40-CAT). (**C, D**) Basal and Tax-induced transcriptional activities. HeLa cells were co-transfected with 5 μg of the indicated CAT reporter plasmids (p21-HTLV-CAT, p21-HIV-CAT and p21-SV40-CAT) plus 0.5 μg of pCMV empty vector (w/o Tax) or pIEX (w/ Tax). Basal CAT activity from p21-SV40-CAT-transfected cells was taken as 100% (**C**, column 3).

In the course of our analyses, we noted that Tax can activate the HTLV-I minimal TATAA-promoter without any known enhancer element by approximately 4-fold (Fig. 1D, lane 1). This responsiveness of the HTLV-I minimal promoter is compatible with the concept that the core promoter can also be an important determinant of transcriptional specificity [2]. We next asked whether all TATAA-elements are recognized by Tax in the same way for purposes of activated transcription. Hence, we constructed reporter plasmids that contain two 21-bp repeats and a minimal TATAA promoter from HTLV-I, HIV-1 or SV40 (Fig. 5A). Since the TATAA promoters were all placed within the same context, we consider this a valid comparison of their relative responsiveness to Tax activation.

**Figure 6:**
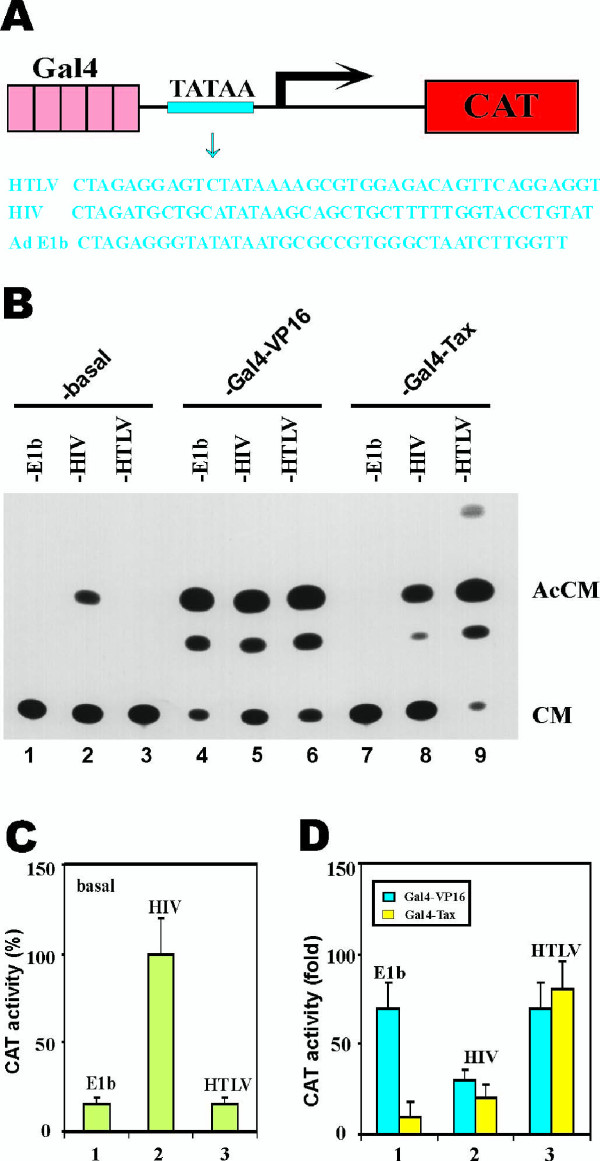
DNA-tethered Tax is specifically active on the HTLV-I minimal promoter. (**A**) CAT reporter plasmid. Each plasmid contains five tandem copies of Gal4-binding sites and one copy of minimal promoter (TATAA) from adenovirus E1b, HIV-1 and HTLV-I. The minimal promoter sequences are shown in blue. (**B**) A representative example of CAT assay. The cells were co-transfected with 2 μg of a Gal4DB plasmid (pM vector alone for lanes 1–3, pGal4-VP16 for lanes 4–6, and pGal4-Tax for lanes 7–9) and 5 μg of a CAT reporter construct (pG5-E1B-CAT for lanes 1, 4 and 7; pG5-HIV-CAT for lanes 2, 5 and 8; and pG5-HTLV-CAT for lanes 3, 6 and 9). (**C, D**) Basal and activated transcriptional activities. HeLa cells were co-transfected with 5 μg of the indicated CAT reporter plasmids (pG5-E1B-CAT, pG5-HIV-CAT and pG5-HTLV-CAT) plus 2 μg of pM empty vector (**C**), pGal4-VP16 (**D**, blue) or pGal4-Tax (**D**, yellow). Basal CAT activity from pG5-HIV-CAT-transfected cells was taken as 100% (**C**, column 2).

While the basal activities of HIV-1 and SV40 minimal promoters were measurably greater than that from HTLV-I (Fig. 5C), replacement of the HTLV-I TATAA with the counterpart element from either HIV-1 or SV40 led to a significant reduction in Tax responsiveness (Fig. 5B, lanes 4–9; and Fig. 5D). To further verify the importance of the TATAA-promoter, we asked the same question using a different approach. Above, Tax was recruited presumably to the downstream TATAA-box via factors bound to the HTLV-1 21bp repeats (see Fig. 5A). We next investigated whether the same conclusion could be established if a Gal4 DNA-binding domain-Tax fusion protein (Gal4-Tax) was delivered to downstream TATAA element by tethering to upstream Gal4-binding sites (see Fig. 6A for reporter schematic). For this assay, we tested the HTLV-I, the HIV-1, and the E1b TATAA-elements. Consistent with the results from the 21 bp-TATAA experiments (Fig. 5), Gal4-Tax activated most strongly the HTLV-I TATAA element (Fig. 6B, lane 9 and Fig. 6D, group 3) and was minimally potent for the adenoviral E1b promoter (Fig. 6B, lane 7 and Fig. 6D, group 1). As a control for Gal4-Tax, we checked in parallel the activity of the artificial Gal4-VP16 activator. In contrast with Gal4-Tax, Gal4-VP16 showed no preference for the various TATAA elements (Fig. 6B, lanes 4–6 and Fig. 6D). Thus, two lines of evidence here support that the HTLV-I TATAA promoter is an additional Tax-specific responsive element.

### Evidence for Tax activity after assembly of an initiation complex

Artificial recruitment of TBP to some higher eukaryotic promoters bypasses transcriptional activation by a DNA-tethered activator [21-24]. When observed at such promoters, this finding is evident that those activators act mechanistically to enhance TBP recruitment to the TATAA box. For general transcriptional activation, additional events subsequent to TBP recruitment are also known to be functionally critical [21-23,25]. To date, it remains unclear whether Tax works transcriptionally through a mechanism solely to recruit TBP or whether there are additional mechanistic implications after TBP is recruited to the TATAA-element.

**Figure 7:**
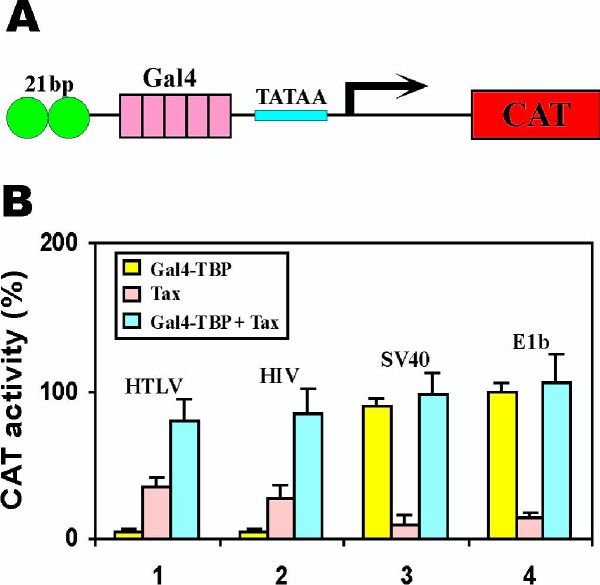
Tax further activates a promoter with DNA-tethered TBP. (**A**) CAT reporter plasmid. Each plasmid contains two copies of 21-bp repeat, five copies of Gal4-binding sites and one copy of minimal promoter (TATAA) from adenovirus HTLV-I, HIV-1, SV40 and adenovirus E1b. (**B**) CAT assay. HeLa cells were co-transfected with 5 μg of the indicated CAT reporter plasmids (p21-G5-HTLV-CAT, p21-G5-HIV-CAT, p21-G5-SV40-CAT and p21-G5-E1B-CAT) and 2 μg of pGal4-TBP (yellow) or 2 μg of pIEX (Tax; pink) or 2 μg of pGal4-TBP plus 2 μg of pIEX (Gal4-TBP + Tax; blue). Basal CAT activity from cells transfected with pGal4-TBP plus p21-G5-E1B-CAT was taken as 100% (group 4, yellow).

To investigate the mechanism(s) of Tax function with respect to TBP recruitment, we constructed a series of reporter plasmids (Fig. 7A) with two copies of 21-bp repeat, five copies of Gal4-binding sites and a minimal TATAA sequence from one of four viral promoters (HTLV-I, HIV-1, SV40 and E1b). We artificially delivered TBP to each promoter by provision of Gal4-TBP, and we asked whether Tax has an additional transcriptional effect which is independent of TBP-recruitment to the TATAA-element. If Tax were to serve only for TBP-recruitment, then when TBP is tethered to the TATAA via Gal4-TBP one should expect to see no transcriptional enhancement from Tax. Provocatively, for both the HTLV-I and HIV-1 TATAA elements, Tax stimulated reporter expression greatly over that already achieved with Gal4-TBP (Fig. 7, groups 1 and 2). Consistent with above findings, the SV40 and E1b TATAA elements appear to be transcriptionally rate-limited by TBP recruitment, and Tax has minimal activity on these promoters. However, the findings from the HTLV-I and the HIV-1 reporters provide evidence that more than simply accelerating TBP recruitment Tax can serve transcriptional function(s) subsequent to TBP (TFIID) assembly at the core promoter. This is the first time that Tax has been shown to have a role subsequent to transcriptional initiation complex formation at the promoter.

## Discussion

Here, we have delineated functional requirements for both the TATAA promoter and the 21-bp enhancer elements in HTLV-I Tax mediated activation of the viral LTR. To date Tax has been considered solely to initiate transcription. Our study shows for the first time that Tax has a transcriptional role after assembly of an initiation complex at the promoter.

### Preferential requirements for 21-bp repeats, CREB, and the HTLV-I TATAA box

HTLV-I is etiologically associated with adult T-cell leukemia [38,39]. Expression of Tax leads to immortalization of T lymphocytes [40-42] and transformation of rat fibroblasts [43,44]. Tax is a transcriptional activator that can interact pleiotropically with several different enhancers. In addition to the HTLV-I 21-bp repeats, κB and SRE elements can also mediate Tax activation [4-6]. Amongst these three enhancers, it is clear that the viral 21-bp repeats are the most highly responsive to Tax-activation (Fig. 1D). However, data elsewhere have raised questions as to the identity of the 21-bp binding bZIP factor which is best used to mediate Tax activation [30]. In direct comparisons, we have used matched A-CREB, A-Jun, A-ATF4 and A-LZIP dominant negative mutants to ask which bZIP factor is most contributory to Tax activation. In our cell system, a novel bZIP factor called LZIP [36] can apparently participate in LTR transcription; however, for Tax activation CREB is preferred over ATF4 or c-Jun (Fig. 4).

Beyond the requirement for the 21-bp enhancer, our experiments revealed that the HTLV-I TATAA is also specifically preferred by Tax (Fig. 5 and Fig. 6). This finding is consistent with the general notion that core promoters can contribute specificity to transcriptional regulation [2]. Indeed, core promoter preference by other cellular and viral activators such as Sp1, VP16 and Tat have been documented previously [45-47]. However, the reasons underlying core promoter preferences are poorly understood. TAFs have been suggested to be responsible for the core promoter selectivity of some activators [48-50]. In this vein, the interaction of Tax with TBP [11] and TBP-associated factors such as TAF_II_28 [51] might provide mechanistic explanations.

### Roles of Tax subsequent to TBP recruitment

A provocative notion which emerges from our study is that Tax can further activate a promoter at which TBP has already been artificially tethered (Fig. 7). Experiments in yeast and mammalian cells indicate that many genes can be activated through artificial recruitment of TBP and other components of the basal transcription machinery to their promoters [52,53]. In yeast, artificial recruitment of TBP bypasses the effect of DNA-tethered activators whereas the activators fail to activate transcription when physically fused to components of the basal transcription machinery [54]. This and other lines of evidence support the notion that activator-dependent recruitment of TBP and basal transcription machinery is a major mechanism for transcriptional activation in yeast cells [54,55]. In contrast, artificial recruitment of TBP to mammalian promoters has not yet been extensively studied. Among the few promoters examined, some such as the ones from E1b and thymidine kinase genes can be fully activated by artificially recruited TBP, while others such as HIV-1 and c-fos promoters are stimulated weakly [21-25]. On the other hand, some activators such as VP16, E1A, Tat, E2F1 and IE2 work synergistically with artificially recruited TBP, while others such as Sp1 cannot further enhance the activity of DNA-tethered TBP [21,22]. Thus, artificial recruitment of TBP might insufficiently activate transcription in mammalian cells and different activators might function at different steps with respect to TBP recruitment. Our results indicate that DNA-bound TBP can activate HTLV-I LTR only weakly, but its activity is further enhanced by Tax (Fig. 6). While such experimental results do not exclude that under physiological circumstances the primary function of Tax may be to enhance initiation complex formation (i.e. TBP-recruitment), they do indicate that Tax has an additional transcriptional activity that extends to phases after transcriptional initiation. Currently, we do not know whether this is at the step of promoter clearance, transcriptional elongation, or some other processes. However, we do believe that Tax should be added to the list of mammalian activators that can function at steps subsequent to TBP recruitment [21-25].

All the transcriptional assays in the present study were based on transiently transfected reporters. We noted that transiently transfected and stably integrated promoters might behave differently [24,56]. Obviously, chromatin structure and copy numbers can account for significant differences [56,57]. Future experiments are required to verify whether the observations established here also hold for stably integrated HTLV-I LTRs.

## Methods

### Plasmids

Chloramphenicol acetyltransferase (CAT) reporter plasmid pG5CAT was from Clontech. CAT plasmid pU3RCAT containing the HTLV-I LTR has been previously described [13]. Other CAT plasmids were derived from pCAT-basic (Promega). For each construct, one copy of a minimal promoter and two copies of an enhancer were chemically synthesized and cloned into pCAT-basic. For example, pCRE-HTLV-CAT contains two copies of canonical CRE motif plus one copy of HTLV-I minimal promoter (Fig. 1A). Five copies of Gal4-binding sites as in pG5CAT were also inserted in some reporters. All constructs have the same spacing between the TATAA box and the CAT open reading frame (44 bp) or between the enhancer and the TATAA box (23 bp).

Sequences of canonical CRE, Sp1, AP1 and κB motifs in the reporter plasmids have been described [36,58,59]. HTLV-I 21-bp repeats and serum response element (SRE) in the plasmids were derived from the following synthetic oligonucleotides: 21-bp repeats, 5'-AGCTTAGGCC CTGACGTGTCCCCCTGGATCCTAGGCCCTGACGTGTCCCCCTA-3' and 5'-AGCTTAG GGGGACACGTCAGGGCCTAGGATCCAGGGGGACACGTCAGGGCCTA-3'; SRE, 5'-AGCTACCATATTAGGATCCATATTAGGT-3' and 5'-AGCTACCTAATATGGATCCTAATATGGT-3'. Sequences of the minimal promoter elements from HTLV-I, HIV-1, SV40 and adenoviral E1b have been described [60]. The SV40 early promoter naturally used for expression of the viral T/t antigens was used.

Expression plasmids for wild type and mutant Tax have been described elsewhere [26,61]. pIEX is a Tax expression vector driven by cytomegalovirus IE promoter. Tax mutants are indicated by the amino acid to be changed, the position of the residue, and the replacement amino acid (e.g. Tax S150A). Amino acids that were removed in mutants are indicated as in Tax Δ3–6. Expression vector pM for Gal4 DNA binding domain (Gal4DB; amino acids 1–147) was from Clontech. Tax, human TBP and the activation domain of VP16 fused to Gal4DB were designated Gal4-Tax, Gal4-TBP and Gal4-VP16, respectively. Expression plasmids for Gal4-Tax and Gal4-TBP have been described [10,21]. Expression plasmid for Gal4-VP16 was from Clontech.

Expression plasmid pRSV-KCREB for the dominant-negative CREB protein KCREB [32] was kindly provided by Dr. Richard Goodman. Expression plasmids pCMV-ACREB and pCMV-AFOS for dominant-negative CREB and AP1 proteins A-CREB [33] and A-Fos [34] were gifts from Dr. Charles Vinson. Expression plasmid pCMV-TAM67 for dominant-negative c-Jun protein TAM67 [35] was from Dr. Michael Birrer. Expression plasmids pCMV-AATF4 and pCMV-ALZIP for dominant-negative ATF4 and LZIP proteins A-ATF4 and A-LZIP were derived from pCMV500 provided by Dr. Charles Vinson [33,37]. A-ATF4 contains 304–352 amino acids of human ATF4 and A-LZIP contains 175–223 amino acids of human LZIP. A-ATF4 and A-LZIP can specifically and dominantly inhibit the CRE-binding and CRE-activating activities of ATF4 and LZIP, respectively, in electrophoretic mobility shift assay and CAT reporter assay (data not shown). Expression plasmid for dominant-negative IKKβ (IKKβ DN) was a gift from Dr. Michael Karin [62].

### Reporter assay

HeLa cells were grown in Dulbecco's modified Eagle's medium supplemented with fetal calf serum and antibiotics, seeded at 5 × 10^5 ^cells/well into six-well culture plates and transfected using calcium phosphate method as described [13]. Jurkat cells were cultured in RPMI 1640 medium and transfected by FUGENE 6 reagents (Roche). CAT activity was assayed as previously described [63]. Briefly, transfected cells were harvested and lysed by freezing and thawing. Protein concentration of clarified lysates was determined by Bradford reagent (Bio-Rad). Equal amounts of lysates were mixed with ^14^C-labeled chloramphenicol (Amersham) and acetyl coenzyme A (Calbiochem) for CAT reaction. CAT activities were detected using thin-layer chromatography and quantified by phosphorimager (Molecular Dynamics). For transfection of cells, each well received the same dose of plasmids. The empty vector or pUC19 was added to compensate for the different amounts of plasmids when necessary.

## Competing interests

None declared.

## References

[B1] Lee TI, Young RA (2000). Transcription of eukaryotic protein-coding genes. Annu Rev Genet.

[B2] Smale ST, Kadonaga JT (2003). The RNA polymerase II core promoter. Annu Rev Biochem.

[B3] Flint J, Shenk T (1997). Viral transactivating proteins. Annu Rev Genet.

[B4] Jeang KT, Boros I, Brady J, Radonovich M, Khoury G (1988). Characterization of cellular factors that interact with the human T-cell leukemia virus type I p40x-responsive 21-base-pair sequence. J Virol.

[B5] Ballard DW, Bohnlein E, Lowenthal JW, Wano Y, Franza BR, Greene WC (1988). HTLV-I tax induces cellular proteins that activate the κB element in the IL-2 receptor α gene. Science.

[B6] Fujii M, Sassone-Corsi P, Verma IM (1988). c-fos promoter trans-activation by the tax1 protein of human T-cell leukemia virus type I. Proc Natl Acad Sci USA.

[B7] Kimzey AL, Dynan WS (1998). Specific regions of contact between human T-cell leukemia virus type I Tax protein and DNA identified by photocross-linking. J Biol Chem.

[B8] Lenzmeier BA, Giebler HA, Nyborg JK (1998). Human T-cell leukemia virus type 1 Tax requires direct access to DNA for recruitment of CREB binding protein to the viral promoter. Mol Cell Biol.

[B9] Zhao LJ, Giam CZ (1991). Interaction of the human T-cell lymphotrophic virus (HTLV) type I transcriptional activator Tax with cellular factors that bind specifically to the 21-base-pair repeats in the HTLV-I enhancer. Proc Natl Acad Sci USA.

[B10] Semmes OJ, Jeang KT (1995). Definition of a minimal activation domain in human T-cell leukemia virus type I Tax. J Virol.

[B11] Caron C, Rousset R, Béraud C, Moncollin V, Egly JM, Jalinot P (1993). Functional and biochemical interaction of the HTLV-I Tax1 transactivator with TBP. EMBO J.

[B12] Tie F, Adya N, Greene WC, Giam CZ (1996). Interaction of the human T-lymphotropic virus type 1 Tax dimer with CREB and the viral 21-base-pair repeat. J Virol.

[B13] Jin DY, Jeang KT (1997). HTLV-I Tax self-association in optimal trans-activation function. Nucl Acids Res.

[B14] Basbous J, Bazarbachi A, Granier C, Devaux C, Mesnard JM (2003). The central region of human T-Cell leukemia virus type 1 Tax protein contains distinct domains involved in subunit dimerization. J Virol.

[B15] Wagner S, Green MR (1993). HTLV-1 Tax protein stimulation of DNA binding of bZIP proteins by enhancing dimerization. Science.

[B16] Baranger AM, Palmer CR, Hamm MK, Giebler HA, Brauweiler A, Nyborg JK, Schepartz A (1995). Mechanism of DNA binding enhancement by the HTLV-I transactivator Tax. Nature.

[B17] Huang GJ, Zhang ZQ, Jin DY (2002). Stimulation of IKK-γ oligomerization by the T-cell leukemia virus oncoprotein Tax. FEBS Lett.

[B18] Kwok RP, Laurance ME, Lundblad JR, Goldman PS, Shih HM, Connor LM, Marriott SJ, Goodman RH (1996). Control of cAMP-regulated enhancers by the viral transactivator Tax through CREB and the co-activator CBP. Nature.

[B19] Jiang H, Lu H, Schiltz RL, Pise-Masison CA, Ogryzko VV, Nakatani Y, Brady JN (1999). PCAF interacts with tax and stimulates tax transactivation in a histone acetyltransferase-independent manner. Mol Cell Biol.

[B20] Harrod R, Kuo YL, Tang Y, Yao Y, Vassilev A, Nakatani Y, Giam CZ (2000). p300 and p300/cAMP-responsive element-binding protein associated factor interact with human T-cell lymphotropic virus type-1 Tax in a multi-histone acetyltransferase/ activator-enhancer complex. J Biol Chem.

[B21] Xiao H, Lis JT, Jeang KT (1997). Promoter activity of Tat at steps subsequent to TATA-binding protein recruitment. Mol Cell Biol.

[B22] Majello B, Napolitano G, De Luca P, Lania L (1998). Recruitment of human TBP selectively activates RNA polymerase II TATA-dependent promoters. J Biol Chem.

[B23] Nevado J, Gaudreau L, Adam M, Ptashne M (1999). Transcriptional activation by artificial recruitment in mammalian cells. Proc Natl Acad Sci USA.

[B24] Dorris DR, Struhl K (2000). Artificial recruitment of TFIID, but not RNA polymerase II holoenzyme, activates transcription in mammalian cells. Mol Cell Biol.

[B25] Kim JM, Hong Y, Jeang KT, Kim S (2000). Transactivation activity of the human cytomegalovirus IE2 protein occurs at steps subsequent to TATA box-binding protein recruitment. J Gen Virol.

[B26] Semmes OJ, Jeang KT (1992). Mutational analysis of human T-cell leukemia virus type I Tax: regions necessary for function determined with 47 mutant proteins. J Virol.

[B27] Yoshimura T, Fujisawa JI, Yoshida M (1990). Multiple cDNA clones encoding nuclear proteins that bind to the *tax*-dependent enhancer of HTLV-1: all contain a leucine zipper structure and basic amino acid domain. EMBO J.

[B28] Franklin AA, Kubik MF, Uittenbogaard MN, Brauweiler A, Utaisincharoen P, Matthews MA, Dynan WS, Hoeffler JP, Nyborg JK (1993). Transactivation by the human T-cell leukemia virus Tax protein is mediated through enhanced binding of activating transcription factor-2 (ATF-2), ATF-2 response and cAMP element-binding protein (CREB). J Biol Chem.

[B29] Reddy TR, Tang H, Li X, Wong-Staal F (1997). Functional interaction of the HTLV-1 transactivator Tax with activating transcription factor-4 (ATF4). Oncogene.

[B30] Gachon F, Thebault S, Peleraux A, Devaux C, Mesnard JM (2000). Molecular interactions involved in the transactivation of the human T-Cell leukemia virus type 1 promoter mediated by Tax and CREB-2 (ATF-4). Mol Cell Biol.

[B31] Jeang KT, Chiu R, Santos E, Kim SJ (1991). Induction of the HTLV-I LTR by Jun occurs through the Tax-responsive 21-bp elements. Virology.

[B32] Walton KM, Rehfuss RP, Chrivia JC, Lochner JE, Goodman RH (1992). A dominant repressor of cyclic adenosine 3',5'-monophosphate (cAMP)-regulated enhancer-binding protein activity inhibits the cAMP-mediated induction of the somatostatin promoter *in vivo*. Mol Endocrinol.

[B33] Ahn S, Olive M, Aggarwal S, Krylov D, Ginty D, Vinson C (1998). A dominate-negative inhibitor of CREB reveals that it is a general mediator of stimulus-dependent transcription of c-fos. Mol Cell Biol.

[B34] Olive M, Krylov D, Echlin DR, Gardner K, Taparowsky E, Vinson C (1997). A dominant negative to activation protein-1 (AP1) that abolishes DNA binding and inhibits oncogenesis. J Biol Chem.

[B35] Brown PH, Alani R, Preis LH, Birrer MJ (1993). Suppression of oncogene-induced transformation by a deletion mutant of c-jun. Oncogene.

[B36] Jin DY, Wang HL, Zhou Y, Chun ACS, Kibler KV, Hou YD, Kung H, Jeang KT (2000). Hepatitis C virus core protein-induced loss of LZIP function correlates with cellular transformation. EMBO J.

[B37] Vinson C, Myakishev M, Acharya A, Mir AA, Moll JR, Bonovich M (2002). Classification of human B-ZIP proteins based on dimerization properties. Mol Cell Biol.

[B38] Matsuoka M (2003). Human T cell leukemia virus type I and adult T-cell leukemia. Oncogene.

[B39] Azran I, Schavinsky-Khrapunsky Y, Aboud M (2004). Role of Tax protein in human T-cell leukemia virus type-I leukemogenicity. Retrovirology.

[B40] Grassmann R, Dengler C, Muller-Fleckenstein I, Fleckenstein B, McGuire K, Dokhelar MC, Sodroski JG, Haseltine WA (1989). Transformation to continuous growth of primary human T lymphocytes by human T-cell leukemia virus type I X-region genes transduced by a herpesvirus saimiri vector. Proc Natl Acad Sci USA.

[B41] Akagi T, Ono H, Shimotohno K (1995). Characterization of T cells immortalized by Tax1 of human T-cell leukemia virus type 1. Blood.

[B42] Kasai T, Jeang KT (2004). Two discrete events, human T-cell leukemia virus type I Tax oncoprotein expression and a separate stress stimulus, are required for induction of apoptosis in T-cells. Retrovirology.

[B43] Tanaka A, Takahashi C, Yamaoka S, Nosaka T, Maki M, Hatanaka M (1990). Oncogenic transformation by the tax gene of HTLV-I *in vitro*. Proc Natl Acad Sci USA.

[B44] Gatza ML, Watt JC, Marriott SJ (2003). Cellular transformation by the HTLV-I Tax protein, a jack-of-all-trades. Oncogene.

[B45] Emami KH, Navarre WW, Smale ST (1995). Core promoter specificities of the Sp1 and VP16 transcriptional activation domains. Mol Cell Biol.

[B46] Berkhout B, Jeang KT (1992). Functional roles for the TATA promoter and enhances in basal and Tat-induced expression of the human immunodeficiency virus type 1 long terminal repeat. J Virol.

[B47] Southgate CD, Green MR (1995). Delineating minimal protein domains and promoter elements for transcriptional activation by lentivirus Tat proteins. J Virol.

[B48] Green MR (2000). TBP-associated factors (TAF_II_s): Multiple selective transcriptional mediators in common complexes. Trends Biochem Sci.

[B49] Martel LS, Brown HJ, Berk AJ (2002). Evidence that TAF-TATA box-binding protein interactions are required for activated transcription in mammalian cells. Mol Cell Biol.

[B50] Chen Z, Manley JL (2003). Core promoter elements and TAFs contribute to the diversity of transcriptional activation in vertebrates. Mol Cell Biol.

[B51] Caron C, Mengus G, Dubrowskaya V, Roisin A, Davidson I, Jalinot P (1997). Human TAF_II_28 interacts with the human T cell leukemia virus type I Tax transactivator and promotes its transcriptional activity. Proc Natl Acad Sci USA.

[B52] Xiao H, Friesen JD, Lis JT (1995). Recruiting TATA-binding protein to a promoter: transcriptional activation without an upstream activator. Mol Cell Biol.

[B53] Ptashne M, Gann A (1997). Transcriptional activation by recruitment. Nature.

[B54] Keaveney M, Struhl K (1998). Activator-mediated recruitment of the RNA polymerase II machinery is the predominant mechanism for transcriptional activation in yeast. Mol Cell.

[B55] Li XY, Virbasius A, Zhu X, Green MR (1999). Enhancement of TBP binding by activators and general transcription factors. Nature.

[B56] Okada M, Jeang KT (2002). Differential requirements for activation of integrated and transiently transfected human T-cell leukemia virus type 1 long terminal repeat. J Virol.

[B57] Ryan MP, Stafford GA, Yu L, Morse RH (2000). Artificially recruited TATA-binding protein fails to remodel chromatin and does not activate three promoters that require chromatin remodeling. Mol Cell Biol.

[B58] Jin DY, Chae HZ, Rhee SG, Jeang KT (1997). Regulatory role for a novel human thioredoxin peroxidase in NF-κB activation. J Biol Chem.

[B59] Jin DY, Giordano V, Kibler KV, Nakano H, Jeang KT (1999). Role of adaptor function in oncoprotein-mediated activation of NF-κB: HTLV-I Tax interacts directly with IκB kinase γ. J Biol Chem.

[B60] Bucher P, Trifonov EN (1986). Compilation and analysis of eukaryotic POL II promoter sequences. Nucl Acids Res.

[B61] Chun ACS, Zhou Y, Wong CM, Kung H, Jeang KT, Jin DY (2000). Coiled-coil motif as a structural basis for the interaction of HTLV type 1 Tax with cellular cofactors. AIDS Res Hum Retrov.

[B62] Zandi E, Rothwarf DM, Delhase M, Hayakawa M, Karin M (1997). The IκB kinase complex (IKK) contains two kinase subunits, IKKα and IKKβ, necessaryfor IκB phosphorylation and NF-κB activation. Cell.

[B63] Chun ACS, Jin DY (2003). Transcriptional regulation of mitotic checkpoint gene MAD1 by p53. J Biol Chem.

